# Magnitude of hepatitis B and C virus infections and associated factors among patients scheduled for surgery at Hawassa University comprehensive specialized Hospital, Hawassa City, southern Ethiopia

**DOI:** 10.1186/s13104-019-4456-0

**Published:** 2019-07-15

**Authors:** Meseret Taye, Deresse Daka, Anteneh Amsalu, Siraj Hussen

**Affiliations:** 1 School of Laboratory Science, College of Medicine and Health Sciences, Hawassa University, Hawassa, Ethiopia; 2 School of Biomedical and Laboratory Sciences, Gondar University, Gondar, Ethiopia

**Keywords:** Hepatitis B virus, Hepatitis C virus, Surgical patients, Ethiopia

## Abstract

**Objective:**

The aim of this study was to assess the magnitude of HBV and HCV infection and its associated factors among surgical patients at Hawassa University comprehensive specialized Hospital Hawassa City, southern Ethiopia.

**Result:**

In this study, the prevalence of HBsAg and Anti-HCV among patients scheduled for surgery were 9% and 5.5%, respectively. Patients who practiced multiple sexual partner (AOR = 2.58, CI 1.18–5.61), dental procedure (AOR = 4.20, CI 1.87–9.55) and blood transfusion (AOR = 3.84, CI 1.27–11.65) had higher odds of HBV infection and those who had history of surgical procedure (AOR = 6.05: 95% CI 1.59–23.04) and dental procedure (AOR = 3.70: 95% CI 1.40–9.77) had higher odds of HCV infection.

**Electronic supplementary material:**

The online version of this article (10.1186/s13104-019-4456-0) contains supplementary material, which is available to authorized users.

## Introduction

Viral hepatitis is an inflammation of the liver. Five different types of hepatitis viruses (A-E) are responsible for viral hepatitis [1]. Of these, hepatitis B viruses (HBV) and hepatitis C viruses (HCV) are the substantial cause of hepatitis around the globe [2].

HBV and HCV can be transmitted through contact with an infected person’s: blood, semen and body fluids [3]. Both HBV and HCV also transmitted through sexual intercourse and vertically from mother to child [4]. In medical care providing centers it is transmitted due to the reuse of inadequately sterilized medical equipment and through per-cutaneous inoculation [5]. Surgical procedures are also modes transmission and the staff may expose for hepatitis viruses [6]. In developing nation the nosocomial transmission of new HCV infections is a major health problem [7].

HBV and HCV infections causes serious health problem throughout the world. The burden is high in Asia, Africa, southern Europe and Latin America. HBV is highly contagious, 50–100 and 10 times more infectious than human immune deficiency virus (HIV) and HCV, respectively [8]. More than 240 and 150 million populations were affected by chronic liver disease due to HBV and HCV infection, respectively [9, 10].

The prevalence of chronic HBV infection varies widely due to geographical area, and predominant routes of transmission [11]. It varies widely, from high (≥ 8%) to intermediate (2–7%) and low (< 2%). Similarly HCV infection is high, moderate or low endemic when the prevalence is > 3.5%, 1.5–3.5% and < 1.5%, respectively [12].

In sub Saharan Africa the prevalence of HBV carriers ranges from 10 to 20% [13]. Ethiopia is one of the high burden countries with high prevalence of HBsAg (35.8%) and anti-HCV (22.5%) among chronic liver disease [14].

The prevalence of HBsAg and anti-HCV is high in hospitalized surgical patients. The risks of acquiring hepatitis diseases for medical waste cleaners after accidental sharp injuries were 30% for HBV and 3% for HCV [15].

In Ethiopia, particularly in the study site, there was no information concerning sero-prevalence of HBsAg and anti-HCV in patients scheduled for surgery. The finding of this study provides evidence for health planers and healthcare workers to take correct and appropriate intervention before and during handling of surgical patients.

## Main text

### Methods

A Hospital based cross-sectional study was conducted at Hawassa University comprehensive specialized hospital. This study was conducted from January to April 2018.

Patients who were appointed for any surgery during the study period and gave consent and assent to participate were included. And those patients who need urgent care in the ICU, on HIV treatment, took Vaccine for HBV, seriously ill and unable to be during interview were excluded.

The sample size was calculated using single population proportion formula based on the following assumptions; the required sample size (n) is estimated with a confidence level of 95%. And proportion of 50% and precision of 5%. A systematic random sampling method was used to recruit study participants.

Structured and pretested questionnaire was used to collect relevant information though interview, which was prepared by reviewing prior similar studies. The data was collected by two B.Sc. nurses.

About 5 ml of venous blood sample was collected from each study participant. Sera were tested for HBsAg marker using SD BIOLINE Strip Test (Standard Diagnostic Inc., Korea). The sensitivity and specificity of the test kit was > 99%. It also tested for Anti HCV using rapid test kit, (Biotest Biotech, China). The sensitivity and specificity of the Kit was 99.1% and 99.6%, respectively. Similarly it was tested for HBeAg using (HBeAg) (Insight HBeAg rapid test kit, TULIP DIAGNOSTICS, Germany). The sensitivity and specificity of the Kit was 99.1% and 99.6%, respectively.

The questionnaire was pre-tested on 5% of the calculated sample size. All data collectors were trained. The collected data were checked daily. The quality of test results were checked using the internal and external quality control.

Data was analyzed using SPSS version 23 software. Bivariate analysis was performed to select candidate variables. Variables with P-value < 0.25 were nominated for multivariable analysis. P-values < 0.05 was considered statistically significance.

### Results

A total of 422 study participants were enrolled in this study. Of these, 51.2% were males and 48.8% were females. The mean age was 35 years. The majority of the study participants (64.9%) were rural in residence and 27.3% had no formal education. Regarding to marital status (64.7%) was married (Additional file 1: Table S1 and Additional file 2: Table S2).

The overall prevalence of HBV and HCV were 9.0% (38/422) and 5.5% (23/422), respectively. Of these, only one patient (0.20%) was co-infected. Of, the HBs Ag positive cases, 34.2% of patients had evidence of HBeAg in their serum.

Exposure to different factors for HBV and HCV infection were presented in (Table 1). In multivariate analysis, multiple sexual partner (AOR = 2.58, CI 1.18–5.61), dental procedure (AOR = 4.20, CI 1.87–9.55) and blood transfusion (AOR = 3.84, CI 1.27–11.65) remained statistically significant factors associated with HBV infection.

**Table 1:** Bivariate and multivariate analysis of factors associated with HBV infection among patients scheduled for surgery at Hawassa University Comprehensive Specialized Hospital, southern Ethiopia, 2018

Categories	HBsAg				
	No. tested (%)	No. positive (%)	COR (95% CI)	AOR (95% CI)	P-value
Sharing of sharps					
Yes	9 (2.1)	1 (11.1)	1.27 (0.16,10.44)		
No	413 (97.9)	37 (9.0)	1		
Contact with people having liver disease					
Yes	8 (1.9)	2 (25.0)	3.50 (0.68,17.98)	3.72 (0.67,20.59)	0.133
No	414 (98.1)	36 (8.7)	1	1	
History of hospital admission					
Yes	33 (7.8)	8 (24.2)	3.83 (1.59,9.22)	2.64 (0.97,7.18)	0.058
No	389 (92.2)	30 (7.7)	1	1	
History of multiple sexual partners					
Yes	76 (18.0)	13 (17.1)	2.65 (1.29,5.46)	2.58 (1.18,5.61)	0.017
No	346 (82.0)	25 (7.2)	1	1	
History of blood transfusion					
Yes	22 (5.2)	6 (27.3)	4.05 (1.49,10.99)	3.84 (1.27,11.65)	0.017
No	400 (94.8)	32 (8.0)	1	1	
History of surgical procedure					
Yes	21 (5.0)	6 (28.6)	4.61 (1.67,12.71)	2.28 (0.71,7.29)	0.166
No	401 (95.0)	32 (8.0)	1	1	
History of dental procedure					
Yes	51 (12.1)	13 (25.5)	4.74 (2.24,10.01)	4.20 (1.87,9.55)	0.001
No	371 (87.9)	25 (6.7)	1	1	
History of tattooing					
Yes	32 (7.6)	4 (12.5)	1.49 (0.49,4.52)		
No	390 (92.4)	34 (8.7)	1		

*COR* crude odds ratio, *AOR* adjusted odds ratio, *CI* confidence interval, *P-v* p-value, 1 reference

*Candidate variable for multivariate analysis at P < 0.25

In further analysis, the risk of having HCV infection remained significantly higher in patients who had history of surgical procedure (AOR = 6.05: 95% CI 1.59–23.04) and dental procedure (AOR = 3.70: 95% CI 1.40–9.77) as compared to their counterparts (Table 2).

**Table 2:** Bivariate and multivariate analysis of factors associated with HCV infection among patients scheduled for surgery at Hawassa University Comprehensive Specialized Hospital, southern Ethiopia, 2018

Categories	Anti-HCV				
	No. tested (%)	No. positive (%)	COR (95% CI)	AOR (95% CI)	P-value
Sharing of sharps					
Yes	9 (2.1)	1 (11.1)	4.66 (0.93,23.29)	3.37 (0.46,24.87)	0.234
No	413 (97.9)	22 (5.3)	1	1	
Contact with people having liver disease					
Yes	8 (1.9)	1 (12.5)	2.55 (0.30,21.61)		
No	414 (98.1)	22 (5.3)	1		
History of hospital admission					
Yes	33 (7.8)	4 (12.1)	2.69 (0.86,8.42)	1.26 (0.32,4.86)	0.740
No	389 (92.2)	19 (4.9)	1	1	
History of multiple sexual partner					
Yes	76 (18.0)	7 (9.2)	2.09 (0.83,5.28)	1.74 (0.65,4.65)	0.270
No	346 (82.0)	16 (4.6)	1	1	
History of blood transfusion					
Yes	22 (5.2)	3 (13.6)	3.00 (0.82,10.99)	1.84 (0.41,8.34)	0.431
No	400 (94.8)	20 (5.0)	1	1	
History of surgical procedure					
Yes	21 (5.0)	4 (19.0)	4.73 (1.45,15.44)	6.05 (1.59,23.04)	0.008
No	401 (95.0)	19 (4.7)	1	1	
History of dental procedure					
Yes	51 (12.1)	8 (15.7)	4.41 (1.77,11.02)	3.70 (1.40,9.77)	0.008
No	371 (87.9)	15 (4.0)	1	1	
History of tattooing					
Yes	32 (7.6)	5 (15.6)	3.83 (1.32,11.10)	3.09 (0.89,10.75)	0.075
No	390 (92.4)	18 (4.6)	1	1	

### Discussion

In this study, the prevalence of HBsAg and Anti- HCV among patients scheduled for surgery at HUCSH were 9% [95% confidence interval (CI) 6.4–11.8%] and 5.5% [95% confidence interval (CI) 3.3–7.6%], respectively. This makes the study sites as high endemic area for both HBV and HCV infection according to WHO criteria [12]. This was in agreement with results reported in similar study population in Pakistan (9.33%) [16]. In contrast, lower prevalence was reported in a study conducted in Sudan (4.91%) [17].

Although direct comparison is difficult because of difference in study population, the sero-prevalence in the current study was in agreement with a previous study reported among pregnant women in Hawassa Ethiopia with a prevalence of (7.8%) [18], in systematic review and meta-analysis done in Ethiopia: blood donors (8.4%) and community based studies (8.0%) [19]. It is also consistent with a (3.7%) prevalence in a study conducted in Addis Ababa [20] and (5%) in Jimma, Ethiopia [21].

Furthermore, the rate of Anti-HCV among patients scheduled for surgery at HUCSH was in agreement with results reported in similar study population in Pakistan (5.1%) [22]. Higher prevalence were also reported in Pakistan (9.09%) [23]. And lower result was also reported in Iraq (0.4%) [24]. In contrast, unusual high HCV prevalence was reported in Bahir Dar (13.3%) [25]. The difference in the rate of HBsAg and Anti-HCV may be due to diverse risk factors involved in various geographical regions and different diagnostic methods employed. There are studies that used more sensitive laboratory methods (ELISA and PCR) compared to the rapid diagnostic test employed in the present study.

In this study, the prevalence of HBeAg among HBsAg positive individuals was (34.2%). Consistent finding was also reported in Nigeria [26]. The presence of HBeAg in the serum of patients with hepatitis B virus is a reflection of active viral replication in hepatocytes and is considered a surrogate marker for the presence of the DNA [27]. This reflects a pool of individuals who are highly infectious and serve in sustaining viral transmission and evolution in the population and health care providers.

Studies showed that due to sharing of similar transmission route, co-infection with HBV and HCV was common. However, only one patient (0.2%) was co-infected in this study. This finding agree in a study [23]. Co-infection of chronic HBV and HCV seems to result in more severe liver disease and risk of liver cancer [28]. The difference in the magnitude might be attributable to difference in the study population, geographical variation, and difference in methodology.

In this study, patients who had history of dental procedure had a fourfold higher chance of acquiring HBV and HCV infection. This finding was also supported by a study [29]. The higher prevalence in those who practiced might be due to most of the dental practices were done out of health institutions with unhygienic conditions and without proper sterilization.

Surgical patients who practiced sex with multiple sexual partners had higher odds of HBV infection compared to their counterparts. Similar findings was reported in in Nigeria [30]. The high prevalence rate may be due to the fact that, Hepatitis B virus infection is sexually transmitted and the transmission increases with the duration of sexual activity and number of sexual partners.

In this study, surgical patients who had history of blood transfusion had about 3.8 times higher odds of HBV infection, compared to their counterparts. The significant association of having history of blood transfusion with HBV infection was also documented in Nigeria [31]. The possible explanation for significant association between HBV infection and blood transfusion might be lack of improved laboratory screening methods of HBV infection from blood donors before transfusion in Ethiopia.

In this study, patients who had history of surgical procedure had six times higher odds of infection with HCV than patients who had no history of surgical procedure. This finding was similar with study conducted in Pakistan [23]. The may be due to the fact that lack of routine serological screening prior to surgery which is one of the factors responsible for increased disease transmission and re-use of contaminated syringes, surgical instruments and improperly screened blood products [32].

## Conclusion

High prevalence of Hepatitis B and C virus infection was observed among patients scheduled for surgery in the study area. Therefore routine screening of all surgical patients before surgery and surgical procedures could be strictly followed, give health education to the community and minister of Health include screening program for all patients before surgery.

## Limitations

First, screening of only surgical patients for HBV and HCV couldn’t determine the exact rate of transmission from patients to HCW or vice versa. Second, due to budget and lack of facility ELISA, HBcAb tests and PCR tests were not done.

## Additional files

**Additional file 1: Table S1.** Questionnaire: to investigate the risk factor for hepatitis B virus and hepatitis C virus in, Hawassa University comprehensive specialized hospital, Hawassa, Ethiopia.

**Additional file 2.** Distribution of HCV by socio-demographic characteristics of study participants scheduled for surgery at Hawassa, University comprehensive specialized Hospital, southern Ethiopia, 2018.

## Data Availability

There is no remaining data and materials, all information is clearly presented in the main manuscript.

## Abbreviations

HBV: hepatitis B virus
HCV: hepatitis C virus
HCC: hepatocellular carcinoma
HUCSH: Hawassa University comprehensive specialized hospital
ICU: intensive care unit
WHO: World Health Organization
