# Supplementary material for: Magnitude of hepatitis B and C virus infections and associated factors among patients scheduled for surgery at Hawassa University comprehensive specialized Hospital, Hawassa City, southern Ethiopia
Source: BMC Res Notes. 2019 Jul 15;12:412. doi: 10.1186/s13104-019-4456-0 (PMC6632215; doi:10.1186/s13104-019-4456-0)
Supplement: Supplementary file 1 — Additional file 1: Table S1. Questionnaire: to investigate the risk factor for hepatitis B virus and hepatitis C virus in, Hawassa University comprehensive specialized hospital, Hawassa, Ethiopia. [file 13104_2019_4456_MOESM1_ESM.docx]

Table S1: Distribution of HBV by socio-demographic characteristics of study participants scheduled for surgery at Hawassa University comprehensive specialized hospital, Southern Ethiopia, 2018.

| **Variables** | | **HBsAg** | | | | |
| --- | --- | --- | --- | --- | --- | --- |
|  |  | **No. tested** (%) | **No. positive** (%) | **COR** (95% CI) | **AOR**(95% CI) | **P-Value** |
| **Sex** | | | | | | |
|  | Female | 216(51.2) | 17(7.9) | 1 |  |  |
|  | Male | 206(48.8) | 21(10.2) | 1.33(0.68,2.59) |  |  |
| **Age(in years)** | | | | | | |
|  | <20 | 30(7.1) | 4(13.3) | 2.55(0.69,9.36) | 3.07(0.78,12.09) | 0.108 |
|  | 20-29 | 205(48.6) | 21(10.2) | 1.89(0.78,4.59) | 2.14(0.82,4.74) | 0.118 |
|  | 30-39 | 123(29.1) | 7(5.7) | 1 | 1 |  |
|  | 40 and above | 64(15.2) | 6(9.4) | 1.71(0.55,5.33) | 1.38(0.39,4.74) | 0.614 |
| **Residence** | | | | | | |
|  | Urban | 148(35.1) | 12(8.1) | 1 |  |  |
|  | Rural | 274(64.9) | 26(9.5) | 1.19(0.58,2.43) |  |  |
| **Marital Status** | | | | | | |
|  | Single | 83(19.7) | 5(6.0) | 1 |  |  |
|  | Married | 273(64.7) | 26(9.5) | 1.64(0.61,4.42) |  |  |
|  | Separated | 57(13.5) | 6(10.5) | 1.84(0.53,6.33) |  |  |
|  | Widowed & Divorced | 9(2.1) | 1(11.1) | 1.95(0.20,18.82) |  |  |
| **Educational Status** | | | | | | |
|  | No formal education | 115(27.3) | 12(10.4) | 1.37(0.62,3.03) |  |  |
|  | Primary education | 191(45.3) | 15(7.9) | 1 |  |  |
|  | Secondary and above | 116(27.5) | 11(9.5) | 1.23(0.54,2.78) |  |  |
| **Occupation** | | | | | | |
|  | Employed | 86(20.4) | 11((12.8) | 2.18 (0.77, 6.16) |  |  |
|  | Unemployed | 202(47.9) | 17(8.4) | 1.36 (0.52, 3.58) |  |  |
|  | Merchant | 39(9.2) | 4(10.3) | 1.69 (0.45, 6.37) |  |  |
|  | Farmer | 95(22.5) | 6(6.3) | 1 |  |  |

**NB:***Candidate variable for multivariate analysis at P<0.25 *variable significant at P<0.05 **COR:** crude odds ratio, **AOR:** adjusted odds ratio, **CI:** confidence interval, **P-V**: p -value, **1:** reference
