# Supplementary material for: Magnitude of hepatitis B and C virus infections and associated factors among patients scheduled for surgery at Hawassa University comprehensive specialized Hospital, Hawassa City, southern Ethiopia
Source: BMC Res Notes. 2019 Jul 15;12:412. doi: 10.1186/s13104-019-4456-0 (PMC6632215; doi:10.1186/s13104-019-4456-0)
Supplement: Supplementary file 2 — Additional file 2. Distribution of HCV by socio-demographic characteristics of study participants scheduled for surgery at Hawassa, University comprehensive specialized Hospital, southern Ethiopia, 2018. [file 13104_2019_4456_MOESM2_ESM.docx]

Table S2: Distribution of HCV by socio-demographic characteristics of study participants scheduled for surgery at Hawassa, University comprehensive specialized Hospital, Southern Ethiopia, 2018.

| **variables** | | **Anti-HCV** | | | | |
| --- | --- | --- | --- | --- | --- | --- |
|  |  | **No. tested** (%) | **No. positive** (%) | **COR** (95% CI) | **AOR**(95% CI) | **P-Value** |
| **Sex** | |  |  |  |  |  |
|  | Female | 216(51.2) | 13(6.0) | 1.26(0.54,2.93) |  |  |
|  | Male | 206(48.8) | 10(4.9) | 1 |  |  |
| **Age(in years)** | | | | | | |
|  | <20 | 30(7.1) | 4(4.8) | 3.14(0.77,12.89) | 3.32(0.75,14.73) | 0.115 |
|  | 20-29 | 205(48.6) | 14(5.1) | 1 | 1 |  |
|  | 30-39 | 123(29.1) | 4(7.0) | 1.97(0.69,5.57) | 2.12(0.69,6.42) | 0.185 |
|  | 40 and above | 64(15.2) | 2(22.2) | 2.39(0.73,7.83) | 2.52(0.71,8.96) | 0.154 |
| **Residence** | | | | | | |
|  | Urban | 148(35.1) | 9(6.1) | 1.20(0.51,2.85) |  |  |
|  | Rural | 274(64.9) | 14(5.1) | 1 |  |  |
| **Marital Status** | | | | | | |
|  | Single | 83(19.7) | 4(4.8) | 1 |  |  |
|  | Married | 273(64.7) | 14(5.1) | 1.07(0.34,3.33) |  |  |
|  | Separated | 57(13.5) | 4(7.0) | 1.49(0.36,6.22) |  |  |
|  | Widowed & Divorced | 9(2.1) | 1(11.1) | 2.47(0.25,24.84) |  |  |
| **Educational Status** | | | | | | |
|  | No formal education | 115(27.3) | 9(7.8) | 1.94(0.73,5.19) |  |  |
|  | Primary education | 191(45.3) | 8(4.2) | 1 |  |  |
|  | Secondary and above | 116(27.5) | 6(5.2) | 1.25(0.42,3.69) |  |  |
| **Occupation** | | | | | | |
|  | Employed | 86(20.4) | 11((12.8) | 2.18 (0.77, 6.16) |  |  |
|  | Unemployed | 202(47.9) | 17(8.4) | 1.36 (0.52, 3.58) |  |  |
|  | Merchant | 39(9.2) | 4(10.3) | 1.69 (0.45, 6.37) |  |  |
|  | Farmer | 95(22.5) | 6(6.3) | 1 |  |  |

**NB:***Candidate variable for multivariate analysis at P<0.25 *variable significant at P<0.05 **COR:** crude odds ratio, **AOR:** adjusted odds ratio, **CI:** confidence interval, **P-V**: p -value, **1:** reference
